# Clinical Characteristics and Outcomes of Surgically Resected Solitary Pulmonary Nodules Due to Nontuberculous Mycobacterial Infections

**DOI:** 10.3390/jcm8111898

**Published:** 2019-11-07

**Authors:** Yeonseok Choi, Byung Woo Jhun, Jhingook Kim, Hee Jae Huh, Nam Yong Lee

**Affiliations:** 1 Division of Pulmonary and Critical Care Medicine, Department of Medicine, Samsung Medical Center, Sungkyunkwan University School of Medicine, Seoul 06351, Korea; yeonseokc@gmail.com; 2 Department of Thoracic and Cardiovascular Surgery, Samsung Medical Center, Sungkyunkwan University School of Medicine, Seoul 06351, Korea; jhingookkim@gmail.com; 3 Department of Laboratory Medicine and Genetics, Samsung Medical Center, Sungkyunkwan University School of Medicine, Seoul 06351, Korea; pmhhj77@gmail.com (H.J.H.); micro.lee@samsung.com (N.Y.L.)

**Keywords:** nontuberculous mycobacterium, nodule, surgery, treatment

## Abstract

Background: Limited data are available regarding the detailed characteristics and outcomes of surgically resected nontuberculous mycobacterial (NTM) granulomas. Methods: We evaluated the characteristics of 49 NTM granulomas presenting as solitary pulmonary nodules (SPNs) between January 2007 and December 2016. Results: Twenty-five patients (51%) were male and 27 (55%) were never-smokers. Seven (14%) patients had a history of tuberculosis. More than half (51%) of patients were asymptomatic. On chest computed tomography, the median SPN diameter was 18 mm, and approximately half of all SPNs (49%) were located in the upper lobes on chest computed tomography. NTM strain were preoperatively isolated from sputum (46%, 12/26), bronchial wash fluid (54%, 14/26), and needle biopsy specimens (12%, 3/26). *Mycobacterium avium* (71%, 22/31) was the organism most commonly isolated, followed by *Mycobacterium intracellulare* (16%, 5/31). Postoperative pneumothorax and atelectasis developed in four (8%) patients and one (2%) patient, respectively. Five patients received postoperative antibiotic therapy. Over a median follow-up period of 18.0 months, one patient with residual lesions after surgery started macrolide-based therapy due to aggravated symptoms. Conclusions: Most NTM granulomas can be treated completely by surgical resection without antibiotic therapy, and microbiological examination of surgical specimens is important for optimal management.

## 1. Introduction

Non-tuberculous mycobacteria-pulmonary disease (NTM-PD) is a chronic progressive infectious disease, and its burden is rapidly increasing worldwide [1,2]. Although the major causative organisms of NTM-PD differ by geographical region, the most common pathogens include *Mycobacterium avium* complex (including *M. avium* and *M. intracellulare*), *M. abscessus* (including *M. abscessus* subsp. *abscessus* and *M. abscessus* subsp. *massiliense*), and *M. kansasii* [3]. NTM-PD has heterogeneity with regard to radiologic findings, but it traditionally has been divided into two phenotypes. The fibrocavitary form is progressive and is characterized by cavitary lesions that are typically in the upper lobes, while the nodular bronchiectatic form presents as bilateral bronchiectasis with nodular infiltrates involving the middle lung zones [4,5].

NTM-PD can also present as solitary pulmonary nodules (SPNs) without cavitary lesions or bronchiectasis [4,6]. Studies have shown that these findings correspond histopathologically to granuloma formation [7,8]. The introduction of chest computed tomography (CT) for lung cancer screening has increased detection of SPNs. SPNs are a common, worrisome clinical problem because they can indicate early-stage lung cancer and are often difficult to non-invasively distinguish NTM SPNs from other inflammatory nodules such as tuberculosis granulomas. Thus, SPNs are frequently surgically resected for definitive diagnosis or treatment.

Current guidelines state that, in the absence of other radiographic evidence of NTM-related disease, surgical resection of the SPN alone may be curative and antibiotic therapy is unnecessary, especially for *M. avium* complex [4,9]. However, these guidelines are primarily based on expert opinion and clinical experience with lung resection for possible neoplasia or lung volume reduction surgery in obstructive lung disease [10], and it is not known if this approach is applicable to NTM species other than *M. avium* complex. There is lack of evidence supporting this approach and limited data on outcomes of NTM SPNs. Therefore, in this study, we evaluated the yield of non-invasive diagnostic modalities for detecting NTM SPNs, and clinico-radiological features and outcomes of surgically resected NTM SPNs.

## 2. Materials and Methods

### 2.1. Study Population

Patients who were diagnosed with a solitary NTM granuloma between January 2007 and December 2016 were retrospectively identified among patients who underwent thoracic surgery for benign lung disease at Samsung Medical Center (1979-bed university-affiliated tertiary referral hospital in South Korea) using medical records. A solitary NTM granuloma was diagnosed as follows: (1) solitary lung nodule defined as a round or oval lesion, on chest CT with no nodular clusters in the same lobe except for lesions adjacent to the main nodule; (2) positive NTM culture from a respiratory specimen such sputum, bronchial washing fluid, or lung biopsy; and (3) histopathologic features of an inflammatory granuloma. Patients who had been treated with chemotherapy before surgery were excluded given the potential effects on pathologic features. Postoperative chemotherapy was prescribed at the discretion of the attending physician. Forty-nine patients who underwent surgical resection of an NTM granuloma were included in the final analysis. The Institutional Review Board of Samsung Medical Center approved the study protocol. Informed consent was waived as this was a retrospective study.

All procedures performed in studies involving human participants were in accordance with the ethical standards of the institutional and/or national research committee and with the 1964 Helsinki declaration and its later amendments or comparable ethical standards. Institutional Review Board of Samsung Medical Center is the local ethics committee that reviewed and approved the protocol. Informed consent was obtained from all individual participants included in the study.

### 2.2. Microbiological Evaluation

Sputum, bronchial washing fluid, or tissue was obtained for microbiological evaluation. Acid-fast bacilli smears and cultures were conducted using standard methods. NTM species were identified using polymerase chain reaction-restriction fragment length polymorphism analysis or reverse-blot hybridization of the *rpoB* gene. Beginning in June 2014, species identification was conducted via nested multiplex polymerase chain reaction and a reverse-hybridization assay of the internal transcribed spacer region (AdvanSureTM Mycobacteria GenoBlot Assay; LG Life Sciences, Seoul, South Korea).

### 2.3. Radiological Evaluation

All patients underwent chest CT within two months prior to surgery. Two of the authors (Y.C. and B.W.J.) reviewed the CT images. The maximal long-axis diameter of the nodule was measured, and CT findings including the presence of calcification, satellite lesion, and location were analyzed. All data with inter-observer disagreement were re-evaluated until consensus was reached. Positron emission tomography with fluorodeoxyglucose (FDG-PET) images were available for 41 patients and maximum standardized uptake values (SUV_max_) were measured [11].

### 2.4. Statistical Analysis

Data are presented as medians (interquartile range (IQR)) for continuous variables or numbers (percentage) for categorical variables. Statistical analysis was performed using SPSS 25.0 (IBM Corp., Armonk, NY, USA).

## 3. Results

### 3.1. Patient Characteristics

Characteristics of the 49 study patients are presented in Table 1. Of them, 25 (51%) were male and 24 (49%) were female with a median age of 59 years (IQR 51–68 years). The median body mass index was 23.4 kg/m^2^ (IQR 21.2–25.1 kg/m^2^) and 27 (55%) patients were never-smokers. Seven (14%) patients had a history of tuberculosis and 13 (27%) patients had a history of solid tumors. More than half (51%) of all patients were asymptomatic and their SPNs were detected at regular checkups or during the course of follow-up for other diseases. The remaining patients presented cough (37%), sputum (29%), or blood tinged sputum/hemoptysis (8%). No patients had a previous history of NTM disease.

### 3.2. Radiological Characteristics of NTM Nodules

**Figure 1:**
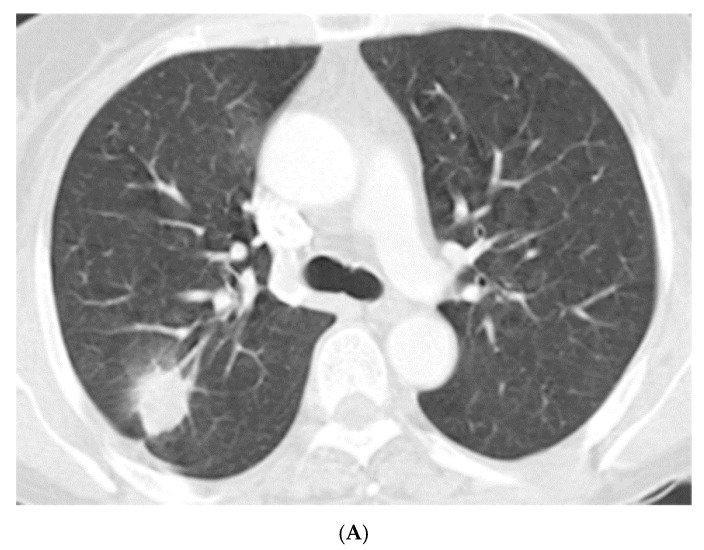

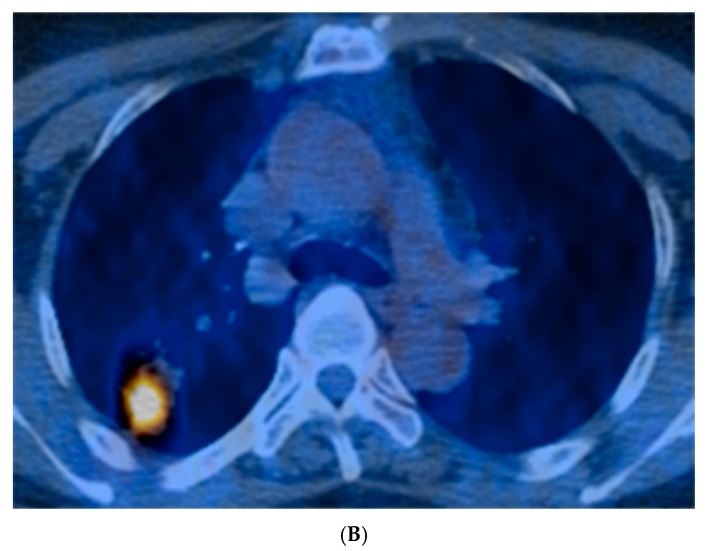
(**A**) A 56-year old female with a solitary pulmonary nodule. Chest computed tomography showed a 23-mm-sized oval nodule at the posterior segment of right upper lobe. (**B**) Positron emission tomography with fluorodeoxyglucose (FDG) revealed a nodule with increased FDG uptake in the right upper lobe mimicking malignancy.

Radiologic characteristics of NTM nodules are summarized in Table 2. The median long-axis diameter of 49 nodules on chest CT was 18.0 mm (IQR 12.5–28.5 mm), and approximately half (49%) were located in the upper lobes. Most (82%) were located in the subpleural area (Figure 1A). Spiculation and pleural invagination were observed at 80% and 73% of the nodules, respectively. About a third of the nodules (31%) had associated calcification and 3 (6%) had satellite lesions. Of the 41 nodules for which FDG-PET/CT was available, the median SUV_max_ was 4.9 (IQR 2.5–8.7), and 76% (31/41) of them had relatively high uptake (SUV_max_ > 2.5) (Figure 1B).

### 3.3. Microbiological Results

Microbiological data of 49 NTM nodules according to diagnostic modality are presented in Table 3. NTMs were isolated from all 49 of the NTM nodules either on preoperative diagnostic workup and/or surgical specimens, and NTM was identified by preoperative workup in 26 (53%) patients. NTMs were isolated in 46% (12/26) of sputum, 54% (14/26) of bronchial washing, and, 12% (3/26) of needle biopsy specimens. When both sputum culture and bronchial washing were performed, the NTM isolation rate was 88% (23/26). NTM was isolated in 59% (29/49) of surgically resected lung specimens.

Species identification was performed in 31 patient specimens (31/49, 63%). *M. avium* (71%, 22/31) was the most commonly isolated, followed by *M. intracellulare* (16%, 5/31). *M. kansasii*, *M. gordonae*, and *M. szulgai* were identified in one (1/31, 3%) patient each. Interestingly, a mixed infection with *M. avium* and *M. kansasii* was identified in one (1/31, 3%) patient.

### 3.4. Treatment Outcomes

**Figure 2:**
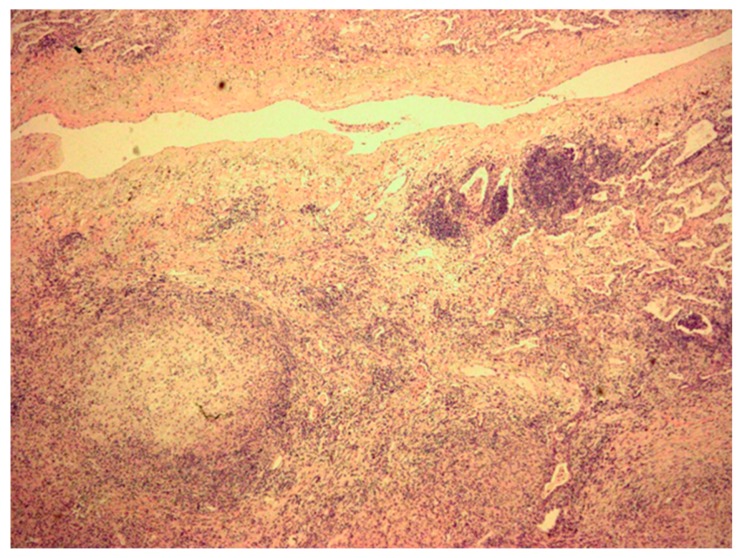
Microscopic findings of video-assisted thoracopscopic surgery biopsy shows granuloma showing a peripheral rim of epithelioid histiocytes surrounding the central necrotic region (hematoxylin-eosin stain, ×40 magnification).

Treatment outcomes are described in Table 4. All patients underwent surgical resection of their nodules, which had typical pathological findings of a granuloma (Figure 2). Most patients (88%, 43/49) underwent wedge resection. The remaining patients with NTM SPNs underwent lobectomy (5/49, 10%) or segmentectomy (1/49, 2%) (ranging from 27 mm to 60 mm, in the six patients). Resection through video-assisted thoracic surgery (96%) was the most common operative approach. Postoperative pneumothorax and atelectasis developed in four (8%) and one (2%) patients(s), respectively, and they recovered without any sequelae.

Five patients received postoperative antibiotic therapy with a median treatment duration of 6.2 months (IQR 4.6–17.6 months); of them, three patients had *M. avium* complex (2 *M. avium* and 1 *M. intracellulare*), and one had *M. kansasii* disease. The remaining one patient had unidentified NTM but treated with macrolide-containing regimen. During a median follow-up period of 18.0 months (IQR 5.0–57.5 months), most (98%, 43/44) of patients who did not receive postoperative antibiotic therapy were clinically stable without complication or recurrence of NTM disease. Only one patient with few residual lesions was started macrolide-based therapy due to aggravated respiratory symptoms for four months after surgery (*M. intracellulare*).

## 4. Discussion

Our data showed that NTM granulomas presenting as SPNs have favorable outcomes. Most cases can be treated completely by surgical resection without perioperative antibiotic therapy. Ninety-eight percent of 44 patients who did not receive postoperative antibiotics were stable without complications or recurrence of NTM disease. Postoperative antibiotic therapy was prescribed in only five of our patients, but baseline characteristics between those who received antibiotics after surgery and those who did not were significantly different (data not shown). These data support the current guidelines [4,9], which indicate surgical resection of a SPN due to NTM may be curative in the absence of other radiographically detected NTM lesions. Similar to our results, some studies also showed favorable outcomes of NTM SPNs. In a Japanese study that evaluated 28 cases of surgically resected NTM SPNs, there was no difference in recurrence between those who received postoperative chemotherapy (*n* = 9) and those who did not (*n* = 19) [12]. A study that compared characteristics of 24 SPNs caused by *M. avium* complex with other granulomas showed that no patients developed new nodules or disseminated disease during follow-up [13]. However, there are still limited data on the natural course of NTM SPNs and optimal treatment, and our data provide insight in this context.

In our study, the rate of NTM species isolation from sputum or bronchial washing fluid was very low, about 50%. These findings suggest that non-invasive testing using respiratory specimens may frequently be non-diagnostic or cannot exclude NTM granulomas, and thus microbiological examinations using tissue specimens are also important for the diagnosis of suspected NTM granuloma. Consistent with our data, other studies of NTM granulomas revealed that preoperative bronchoscopy did not play a significant role in the microbiological diagnosis of NTM SPNs. For example, in a 1981 study that evaluated 20 granulomas presenting as SPNs, bronchoscopy was not diagnostic, but *M. avium* complex was cultured from bronchial washing fluid in only one patient [14]. In a Japanese study in 2006, only 2 of 12 patients with NTM SPNs who underwent bronchoscopy had positive acid-fast bacilli stains, and most cases were confirmed by percutaneous lung biopsy or surgery [15]. In other recent data, positive culture rates from bronchial washing fluid were reported to be as low as 11% to 43% [12,16,17], and the most definitive diagnosis was via histology. However, invasive procedures always present a risk of complications, thus patients should be selected carefully.

In our study, more than three-fourths (76%) of NTM SPNs showed high SUV_max_ uptake on FDG-PET/CT scan, which suggests that FDG-PET/CT is likely not useful for differentiating NTM SPNs from malignant nodules. NTM SPN is a unique phenotype of NTM-PD that is frequently difficult to radiologically distinguish from malignancy. However, FDG-PET can be used to evaluate the glucose metabolism of SPNs and thereby identify an active lesion regardless of whether the lesion is benign or malignant. Granulomatous nodules such as tuberculomas or NTM SPNs can have positive results as shown in our previous study that compared characteristics of tuberculomas and NTM SPNs [7]. Although the criteria for distinguishing malignancy vary among researchers, most NTM SPNs showed relatively high FDG-PET uptake in previous studies [18,19].

Interestingly, we identified rare organisms including *M. kansasii*, *M. szulgai*, and *M. gordonae*, but the most common causative organism was *M. avium*. The reason for this phenomenon is not known, but most previous studies reported similar results. In 1981, Gribetz et al. reported that *M. avium* complex was the most common causative pathogen (60%) in 20 NTM SPNs [14]. In a previous Japanese study, 58% (7/12) of patients had *M. avium* as a causative organism [15]. Recent studies in South Korea also showed that *M. avium* was the causative strain in 82% (9/11) and 58% (7/12) of patients with NTM SPNs, respectively [16,17]. It is well known that NTMs are environmental organisms that inhabit specific niches including natural water sources, especially *M. avium* [20]. However, there is no data explaining whether the high overall frequency of *M. avium* complex among NTM SPN is due to specific bacterial tropism or some other phenomenon. Additionally, given that NTM SPNs caused by rare strains such as *M. kansasii*, *M. gordonae*, *M. fortuitum*, or *M. haemophilum* have been reported [21,22,23,24], further studies of the association between NTM species and clinical features are needed.

There were several limitations in this study. First, this was a single center, retrospective study. However, we included the largest number of patients with surgically resected NTM SPNs as possible. Second, more than 30% of identified nodules did not undergo NTM identification testing, which is why we were unable to evaluate prognosis according to organism. Third, the post-operative follow-up period may not have been long enough to evaluate the long-term prognosis of NTM SPNs.

## 5. Conclusions

In conclusion, the present study suggests that most NTM granulomas can be treated safely by surgical resection without postoperative antibiotic therapy, and microbiological examinations including mycobacterial culture of surgical specimens is important for optimal management of these patients.

## Figures and Tables

**Table 1:** Baseline characteristics of study patients.

Characteristics	Values
Patient number	49 (100)
Sex, male	25 (51)
Age	59 (51–68)
Body mass index (kg/m^2^)	23.4 (21.2–25.1)
Smoking history	
Never	27 (55)
Ex-smoker/current smoker	22 (45)
Prior treatment for tuberculosis	7 (14)
History of solid tumor	13 (27)
Lung cancer	1/13 (8)
Other solid tumors	12/13 (92)
Respiratory symptoms	
Asymptomatic	25 (51)
Cough	18 (37)
Sputum	14 (29)
Blood tinged sputum or hemoptysis	4 (8)

Data are presented as number (%) or median (interquartile range).

**Table 2:** Radiological characteristics of 49 nontuberculous mycobacteria nodules.

Characteristics	Values
Chest computed tomography	
Size (mm) *	18.0 (12.5–28.5)
Location	
Upper	24 (49)
Middle/lingular	7 (14)
Lower	18 (37)
Subpleural nodule	40 (82)
Calcification	15 (31)
Satellite nodules	3 (6)
Spiculation	39 (80)
Pleural invagination	36 (73)
^18^FDG-PET/CT (*n =* 41)	
SUV_max_	4.9 (2.5–8.7)
SUV_max_ (>2.5)	31/41 (76)

Data are presented as number (%) or median (interquartile range). * The maximal long-axis diameter is measured. ^18^FDG-PET, [18F] fluoro-2-deoxy-d-glucose positron emission tomography; CT, computed tomography.

**Table 3:** Microbiological data of 49 nontuberculous mycobacteria nodules.

Characteristics	Values
Modalities for microbiological evaluation	Positive culture rate for NTM
Preoperative workup	26 (53)
Sputum	12/26 (46)
Bronchial washing fluid	14/26 (54)
Needle biopsy specimens	3/26 (12)
Sputum culture AND bronchial washing	23/26 (88)
Sputum culture AND needle biopsy	15/26 (58)
Bronchial washing AND needle biopsy	17/26 (65)
Surgical resection	29 (59)
Identified NTM species	31 (63)
* M. avium*	22/31 (71)
* M. intracellulare*	5/31 (16)
* M. avium* + *M. kansasii*	1/31 (3)
* M. kansasii*	1/31 (3)
* M. gordonae*	1/31 (3)
* M. szulgai*	1/31 (3)
Unidentified, NTM	18 (37)

Data are presented as number (%) or median (interquartile range). NTM, nontuberculous mycobacteria.

**Table 4:** Treatment outcomes of 49 nontuberculous mycobacteria nodules.

Characteristics	Values
Type of surgical resection	
Wedge resection	43 (88)
Segmentectomy	1 (2)
Lobectomy	5 (10)
Operative approach	
Thoracotomy	2 (4)
Video-assisted thoracoscopic surgery	47 (96)
Postoperative complication	5 (10)
Pneumothorax	4 (8)
Atelectasis	1 (2)
Postoperative chemotherapy	5 (10)
ARE	2 (4)
CRE	2 (4)
CRE + HRE	1 (2)
Duration of postoperative chemotherapy (months)	6.2 (4.6–17.6)
Follow up duration (months)	18.0 (5.0–57.5)
Progression to NTM pulmonary disease	1 (2)

Data are presented as number (%) or median (interquartile range). A, azithromycin; R, rifampicin; E, ethambutol; C, clarithromycin; H, isoniazid; NTM, nontuberculous mycobacteria.

## Abbreviations

CT	computed tomography
FDG	fluorodeoxyglucose
IQR	interquartile range
NTM	non-tuberculous mycobacteria
PD	pulmonary disease
PET	positron emission tomography
SPN	solitary pulmonary nodules
SUV_max_	maximum standardized uptake values
